# The *T*rem*2* R47H variant confers loss-of-function-like phenotypes in Alzheimer’s disease

**DOI:** 10.1186/s13024-018-0262-8

**Published:** 2018-06-01

**Authors:** Paul J. Cheng-Hathaway, Erin G. Reed-Geaghan, Taylor R. Jay, Brad T. Casali, Shane M. Bemiller, Shweta S. Puntambekar, Victoria E. von Saucken, Roxanne Y. Williams, J. Colleen Karlo, Miguel Moutinho, Guixiang Xu, Richard M. Ransohoff, Bruce T. Lamb, Gary E. Landreth

**Affiliations:** 1 Department of Neurosciences, Case Western Reserve University, School of Medicine, Cleveland, OH 44106 USA; 2 Department of Anatomy and Cell Biology, Indiana University, School of Medicine, Indianapolis, IN 46202 USA; 3 Paul and Carole Stark Neurosciences Research Institute, Indiana University, School of Medicine, Indianapolis, IN 46202 USA; 4 Department of Medical and Molecular Genetics, Indiana University, School of Medicine, Indianapolis, IN 46202 USA; 5 Cleveland Clinic Lerner Research Institute, Cleveland, OH 44195 USA; 6 Third Rock Ventures, Boston, MA 02116 USA

**Keywords:** TREM2, Neuroinflammation, Innate immunity, CRISPR/Cas9, Single nucleotide polymorphism, Alzheimer’s disease

## Abstract

**Background:**

The R47H variant of Triggering Receptor Expressed on Myeloid cells 2 (TREM2) confers greatly increased risk for Alzheimer’s disease (AD), reflective of a central role for myeloid cells in neurodegeneration. Understanding how this variant confers AD risk promises to provide important insights into how myeloid cells contribute to AD pathogenesis and progression.

**Methods:**

In order to investigate this mechanism, CRISPR/Cas9 was used to generate a mouse model of AD harboring one copy of the single nucleotide polymorphism (SNP) encoding the R47H variant in murine *Trem2*. TREM2 expression, myeloid cell responses to amyloid deposition, plaque burden, and neuritic dystrophy were assessed at 4 months of age.

**Results:**

AD mice heterozygous for the *Trem2* R47H allele exhibited reduced total *Trem2* mRNA expression, reduced TREM2 expression around plaques, and reduced association of myeloid cells with plaques. These results were comparable to AD mice lacking one copy of *Trem2*. AD mice heterozygous for the *Trem2* R47H allele also showed reduced myeloid cell responses to amyloid deposition, including a reduction in proliferation and a reduction in CD45 expression around plaques. Expression of the *Trem2* R47H variant also reduced dense core plaque number but increased plaque-associated neuritic dystrophy.

**Conclusions:**

These data suggest that the AD-associated TREM2 R47H variant increases risk for AD by conferring a loss of TREM2 function and enhancing neuritic dystrophy around plaques.

**Electronic supplementary material:**

The online version of this article (10.1186/s13024-018-0262-8) contains supplementary material, which is available to authorized users.

## Background

Alzheimer’s disease (AD) is accompanied by a robust inflammatory response [1]. However, until recently, it has been unclear whether myeloid cells (including brain-resident microglia and possibly infiltrating monocytes) actively contribute to AD pathogenesis and progression. Recent Genome Wide Association Studies have linked single nucleotide polymorphisms (SNPs) in inflammation-related genes to increased AD risk [2], including a SNP encoding the R47H variant in Triggering Receptor Expressed on Myeloid cells 2 (*TREM2)*. The *TREM2* R47H variant not only constitutes one of the strongest single allele genetic risk factors for AD [3, 4], but also confers elevated risk for Parkinson’s disease, amyotrophic lateral sclerosis, and frontotemporal dementia [5]. Furthermore, homozygous *TREM2* variants cause Nasu-Hakola disease, which is characterized by extensive white matter loss and frontotemporal-like dementia [6]. These genetic studies definitively demonstrate that myeloid cell perturbations can contribute to neurodegenerative disease. However, it remains unclear how the *TREM2* R47H variant alters myeloid cell function to enhance disease risk.

In the brain, TREM2 is expressed exclusively by myeloid cells [7, 8] and has been implicated in a diverse range of myeloid cell functions [5]. A number of studies have investigated the role of TREM2 in AD pathogenesis using *Trem2* deficient mice. Myeloid cells accumulate around amyloid plaques in the AD brain, but the abundance of these plaque-associated myeloid cells is substantially diminished in AD mice lacking *Trem2*, consistent with its known roles in myeloid cell survival and proliferation. Yuan et al. postulate that the loss of plaque-associated myeloid cells promotes plaque expansion and damage to surrounding neurites in *Trem2* deficient mice [9]. In support of this hypothesis, *Trem2* deficient AD mice exhibit enhanced amyloid pathology at late stages in disease [10, 11] accompanied by increased plaque-associated neuritic dystrophy [9, 11, 12]. However, at early stages of disease progression, *Trem2* deficiency reduces amyloid burden [10, 13].

While these studies have elucidated some important aspects of TREM2 function in the context of AD, how these studies relate to disease-associated TREM2 variants has only recently begun to be investigated. In vitro studies have demonstrated that the TREM2 R47H variant reduces affinity for TREM2 ligand binding [9, 11, 14–18], and alters glycosylation [19, 20], leading to speculation that the TREM2 R47H variant may result in a loss of TREM2 function. The function of the R47H variant was recently assessed for the first time in vivo. Song et al. expressed the human *TREM2* R47H variant using a bacterial artificial chromosome (BAC) transgenic and found that the R47H variant could not rescue aspects of TREM2 function in AD mice lacking endogenous *Trem2* expression [21]. This study is in agreement with the in vitro data suggesting the *TREM2* R47H variant results in a loss of TREM2 function. However, because of the approach used in this study, it is unclear whether the loss of function phenotypes observed could be attributed to impairments in association of human TREM2 with mouse signaling pathways. In addition, these mice expressed eight copies of the *TREM2* gene and, because TREM2 overexpression has previously been associated with changes in microglial function and pathology [22], it is difficult to determine which phenotypes observed in this study were due to the TREM2 R47H variant or overexpression of the TREM2 protein. In the current study, we use a complementary approach that maintains endogenous regulation of *Trem2* expression. We address the critical question of how the R47H *Trem2* variant alters TREM2 function in vivo*,* including AD-associated myeloid cell responses, using AD mouse models in which CRISPR/Cas9 was used to knock the R47H variant into the endogenous mouse *Trem2* gene. Using this model, we demonstrate that the *Trem2* R47H variant dramatically reduces TREM2 expression, compromising myeloid cell responses to AD-like amyloid pathology. Furthermore, we are the first to demonstrate that these myeloid cell changes with the R47H *Trem2* variant alter plaque structure to enhance neuritic dystrophy.

## Methods

### Contact for reagent and resource sharing

Further information and requests for resources and reagents should be directed to corresponding authors Gary Landreth (glandret@iu.edu) or Bruce Lamb (btlamb@iu.edu).

### Experimental model

CRISPR/Cas9-mediated insertion of the SNP encoding the TREM2 R47H variant into the mouse *Trem2* gene was performed by injecting embryos with Cas9, short-guide RNA (sgRNA) and replacement oligo. The sequences are as follows: *Trem2* targeted region 3’-CGCAAGGCCTGGTGTCGGCAGCTGGGTGAG, sgRNA (antisense) 5’-CCACAGCCGTCGACCCACTC, and replacement oligo 3’-CACAAGGCTTGGTGTCGGCAGCTGGGTGAG. The first codon in the replacement oligo corresponds to the SNP encoding the R47H variant, while the third codon corresponds to a silent mutation that ablates the protospacer adjacent motif (PAM) site, necessary for initial binding of CRISPR/Cas9. Using Sanger sequencing, mice from six different founder lines were identified to carry the SNP encoding the TREM2 R47H mutation in either heterozygosity or homozygosity. SNP-based genotyping (Thermo Fisher) was used to identify carriers in subsequent crosses using the following: forward primer: 5’-ATGTACTTATGACGCCTTGAAGCA, reverse primer: 5’-ACCCAGCTGCCGACAC, SNP reporter 1: 5’-CCTTGCGTCTCCC, SNP reporter 2: 5’-CCTTGTGTCTCCC.

In order to determine whether off-target mutations occurred with CRISPR/Cas9-targeting, genomic DNA was extracted using the DNeasy Blood and Tissue Kit (Qiagen, 69504) from F1 mice from four independently generated *Trem2* R47H founder lines (R104, R202, R506, and R1019) and independently maintained APPPS1–21; *Trem2*^+/+^ or *Trem2*^+/+^ mice. HiSeqX Sequencing was conducted with at least 30× coverage, 75% of bases above Q30 at 2 × 150 bp (Garvan Institute of Medical Research). Following alignment to the mouse reference genome (MM9), the presence of insertion and deletion mutations were assessed using the variant calling tools GATK-HC and Samtools Mpileup. Sequences are available via http://www.ncbi.nlm.nih.gov under BioProject accession PRJNA471261. CRISPR off-target prediction software (http://www.crispor.tefor.net) was used to determine potential off-target genes in exonic regions of chromosome 17 [23]. Mutations are shown for the only predicted off-target gene *Rab11fip3* and the CRISPR/Cas-9 target (Additional file 1: Table S1). Additionally, in order to address dysregulation of *Trem*-like genes within 5 kb of the *Trem2* locus that may affect myeloid cell function [24], mutations were also assessed in *Treml1, Treml2,* and *Treml6*. Mutations were detected in lines R202 and R506 and these lines were therefore not used for the current study. However, no off-target mutations were identified in R104 or R1019 lines, consistent with the low rate of expected off-target mutations due to CRISPR/Cas9 targeting [25]. These two founder lines were maintained independently and mice from generations F1-F3 were used in the analyses presented here.

*Trem2* deficient mice (*Trem2*^tm1(KOMP)Vlcg^) with replacement of exons 2, 3, and part of 4 with *LacZ* were used to generate *Trem2*^*+/+*^ *and Trem2*^*+/−*^ controls. WT *Trem2* was genotyped using the following primers: forward 5’-TGGTGAGCACACACGGT, reverse 5’-TGCTCCCATTCCGCTTCTT and *LacZ* was genotyped using the following primers: forward 5’-ATCACGACGCGCGCTGTATC, reverse: 5’-ACATCGGGCAAATAATATC. *Trem2*^R47H^ and *Trem2*^tm1(KOMP)Vlcg^ mice were crossed into the APPPS1–21 AD mouse model (kindly provided by Mathias Jucker) which expresses the Swedish APP mutation (KM670/671NL) and the L166P mutation in PSEN1 driven under the *Thy-1* promoter [26]. All mice used in this study were maintained on a C57BL6/J background. Both male and female mice were used in this study.

### Method details

#### Tissue isolation

Following deep anesthetization with ketamine/xylazine, mice were perfused with ice-cold PBS, and brains removed. For immunohistochemistry, one hemisphere was drop-fixed in 4% PFA in PBS for 24–48 h, transferred and stored at 4 °C in 30% sucrose in PBS. After embedding in OCT Compound (VWR), 30 μm thick sections were obtained on a Leica CM 1950 cryostat and stored in cryoprotection buffer containing 30% sucrose, 1% PVP-40, and 30% ethylene glycol in 0.1 M phosphate buffer at − 20 °C until use.

For qPCR and ELISA studies, cortical and hippocampal regions from the other hemisphere were microdissected, snap frozen in liquid nitrogen, and stored at − 80 °C until proceeding to extraction. Tissue was homogenized in buffer containing 1% NP-40, 0.5% sodium deoxycholate, 0.1% SDS, 1:100 protease inhibitor cocktail (Sigma Aldrich, P8340). For Aβ extractions, brain homogenates were stored at − 80 °C. For qPCR, samples were stored in an equal volume of RNA-Bee (Amsbio, CS-104B) at − 80 °C until proceeding to RNA extraction.

#### Quantitative RT-PCR

RNA was isolated using phenol-chloroform extraction and a Purelink RNA Mini Kit (Life Technologies) with an on-column DNAse Purelink Kit (Life Technologies). RNA-to-cDNA conversion was conducted on 500 ng RNA with QuantiTech Reverse Transcription Kit (Qiagen) and qPCR was conducted using a StepOne Real Time PCR System with Taqman Assays (Life Technologies). Gene expression was normalized to *Gapdh* and *18s.* Relative gene expression is graphed as fold change, and ΔCT values were used for statistical analysis. The following genes were assessed: *Arg1* (Mm00475977_m1), *Fizz1* (Mm00445109_m1), *Ym1* (Mm00657889_mH), *Il-1b* (Mm00434228_m1), *Il-6* (Mm00446191_m1), *iNos (*Mm00440502_m1), *Tlr4* (Mm445273_m1), *Tnfa* (Mm443258_m1), and *Trem2* (Mm04209424_g1).

#### Immunohistochemistry

Sections were permeabilized with PBS containing 0.1% Triton X-100. Antigen retrieval was conducted using 10 mM sodium citrate, pH 6.0, with 0.5% Tween-20, except for TREM2 and CD45 for which Reveal Decloaker (Biocare Medical, RV1000) was used. Sections were exposed to antigen retrieval for 10 min at 95 °C, cooled for 20 min, and incubated in blocking buffer containing 5% normal goat serum, 0.3% Triton X-100, in PBS. Sections were incubated in primary antibodies diluted in blocking buffer overnight at 4 °C, washed, and incubated in the appropriate Alexa-Fluor-conjugated secondary antibodies at 1:1000 in blocking buffer for 1 h at room temperature (RT). To detect TREM2, sections were incubated in primary antibody for 48 h at 4 °C and secondary antibody for 6 h at RT. Nuclei were counterstained with DAPI, and slices were mounted and coverslipped with Prolong Gold (Thermo Fisher, P36930). Mouse on Mouse Blocking Reagent (Vector Laboratories, MKB-2213) was used for primary antibodies generated in mouse or rat at 1:1000. TREM2 (R&D Systems, AF1729), 6E10 (Bio Legend 9153–005), IBA1 (Wako, 019–19741), Ki67 (Cell Signaling Technology, RM9106–50), CD45 (ABD Serotec, MCA1388), and n-APP (EMD Milliopore, MAB348) were used at 1:500, and ubiquitin (Thermo Fisher PA1–10023) was used at 1:2000. For dense core plaque staining, sections were washed with PBS, mounted, and stained with 1% *w*/*v* Thioflavin S.

#### Aβ_1–40_ and Aβ_1–42_ ELISAs

Extraction of Aβ species was conducted as described previously [27]. Briefly, cortical tissue-enriched homogenates were combined in equal volume with 0.4% diethylamine (DEA), subjected to ultracentrifugation, and supernatant containing the soluble protein fraction was neutralized with 0.5 M Tris-HCl. The pellet was dissolved in ice-cold 95% formic acid (FA), subjected to ultracentrifugation, and supernatant containing the insoluble protein fraction was neutralized in buffer containing 0.5 M Tris base, 0.5 M Na_2_HPO_4_, and 0.05% NaN_3_. Fractions were stored at − 80 °C until use.

For ELISA detection of Aβ_1–40_ and Aβ_1–42_, F8 Maxisorp Nunc-Immuno Module (Thermo Fisher) wells were coated with 6E10 ascites antibody (Bio Legend, SIG-39300) at 1:1000 diluted in 100 mM carbonate buffer, pH 9.6, overnight at 4 °C. Wells were washed with PBS containing 0.025% Tween-20 and blocked with 1% nonfat milk in PBS for 1 h at 37 °C. DEA fractions, FA fractions, and recombinant Aβ_1–40_ (Bachem, 4014442) and Aβ_1–42_ (Bachem, 4061966) protein standards were diluted in PBS containing 0.025% Tween-20 and 0.5% BSA and incubated overnight at 4 °C. Following incubation with anti-Aβ_1–40_-conjugated HRP (Biolegend, 805407) at 1:2500 or anti-Aβ_1–42_-conjugated HRP (Biolegend, 805507) at 1:1250 for 1 h at RT, Aβ_1–40_ and Aβ_1–42_ were detected using the Pierce TMB Substrate Kit (Thermo Fisher, 34021) and a BioTek Synergy HTX plate reader at 450 nm. Total levels of Aβ were normalized to total protein levels in each fraction using the Pierce BCA Protein Assay Kit (Thermo Fisher, 23225) and values are represented as fold change to APPPS1; *Trem2*^+/+^ animals.

#### Image acquisition

Epifluorescent images for percent area and plaque burden were acquired on a CTR5000 upright epifluorescent microscope (Leica). Confocal images for IBA1-positive cell number were obtained on a LSM 510 META microscope (Zeiss).

### Quantification and statistical analysis

Quantification of all immunohistochemistry experiments was conducted by observers blinded to *Trem2* genotype. Values within one image were averaged together and then averaged for each biological replicate. Data are graphed as the mean ± SEM.

#### Plaque-associated percent area

Plaque-associated percent area of TREM2 and CD45 were assessed using one medial and one lateral matched section per animal. Images were acquired from three cortical regions (motor, somatosensory, and visual cortex) and three hippocampal regions at 20× magnification. A circular ROI centered on 6E10-positive plaques was used to define regions for quantification. Images were manually thresholded and quantified using the Multi-measure ROI function in Image J (NIH).

#### Plaque-associated myeloid cell number

IBA1-positive cell number per plaque was assessed by acquiring confocal Z stacks 0.25 um apart in one medial and one lateral matched section per animal from three cortical regions (motor, somatosensory, and visual cortex) at 20× magnification. Stacks were collapsed into a single image and the number of IBA1-positive cell soma within the ROI centered around 6E10-positive plaques was scored using Image J.

#### Proliferating myeloid cell number

The total number of Ki67, IBA1-double positive cells within one medial and one lateral matched section per animal was manually scored.

#### Plaque burden

For plaque burden, every 12th sagittal section (10–12 sections per animal) was stained with Thioflavin S or 6E10. Images were acquired from three cortical regions (motor, somatosensory, and visual cortex) at 10× magnification and the dorsal hippocampus at 5× magnification per section. 6E10 and Thioflavin S-positive plaque number and area were quantified using the Particle Analysis function in Image J following manual thresholding.

#### Dystrophic Neurite Area & Plaque Size

For the analyses relating plaque size to dystrophic neurite area, images were acquired from three cortical regions (motor, somatosensory, and visual cortex) and three hippocampal regions at 20× magnification from one medial and one lateral matched section per animal. ROIs centered on 6E10-positive plaques were drawn individually for each plaque to include the total area of plaque-associated dystrophic neurites (ubiquitin and n-APP). Following manual thresholding, 6E10 immunoreactive plaque size and dystrophic neurite immunoreactive area for each respective ROI was quantified using the Particle Analysis function in Image J. Dystrophic neurite area was divided by 6E10 plaque size for each plaque. These values were averaged within each image and then across images for each animal to yield the results for dystrophic neurite area / plaque size.

#### Statistical analysis

Prism (Graphpad) was used for all statistical analyses. Grubb’s test with a cutoff of α = 0.05 was used to determine statistical outliers. Statistical significance was determined using a one-way or two-way ANOVA with Bonferroni post hoc analysis, with *p*-values less than 0.05 considered as significant. Each n represents a single biological replicate. Data shown are representative of three independent experiments.

#### Supplemental material

Additional file 2: Figure S1 details the experimental model, main findings in the manuscript across *Trem2* R47H founder lines, *Trem2* expression in non-AD transgene expressing animals, and *Trem2* expression by sex. Additional file 3: Figure S2 details IBA1+ plaque number according to plaque size and the expression of a panel of inflammation-related genes. Additional file 4: Figure S3 shows changes in expression of genes related to Aβ production and Aβ species using ELISA. Additional file 1: Table S1 provides the variant calls for mutations in the off-target predicted gene *Rab11fip3*, *Trem2*, and *Trem*-like genes in mice derived from the first cross from *Trem2* R47H founders.

## Results

To assess whether the *Trem2* R47H variant affects TREM2 expression, myeloid cell function and pathology in AD, we used CRISPR/Cas9 targeting to introduce the G➔A single nucleotide polymorphism (SNP) encoding the variant in the endogenous mouse *Trem2* gene. Successful knock in of *Trem2* R47H was validated using Sanger sequencing (Additional file 2: Figure S1A) and whole genome sequencing did not identify any off-target mutations (Additional file 1: Table S1). Founder lines positive for the SNP were crossed to the APPPS1–21 AD mouse model [26], generating APPPS1–21;*Trem2*^+/R47H^ mice. Mice from two founder lines were maintained independently and generations F1-F3 from both lines were used throughout this study. While working with early generations of these mice does increase the chance that off-target mutations are present, we observed no significant differences in phenotype between the two independent lines (Additional file 2: Figure S1B). These mice were compared to APPPS1–21;*Trem2*^*+/+*^ and APPPS1–21;*Trem2*^*+/−*^ at 4 months of age.

### *Trem2* R47H impairs the myeloid cell response to amyloid pathology

**Fig. 1:**
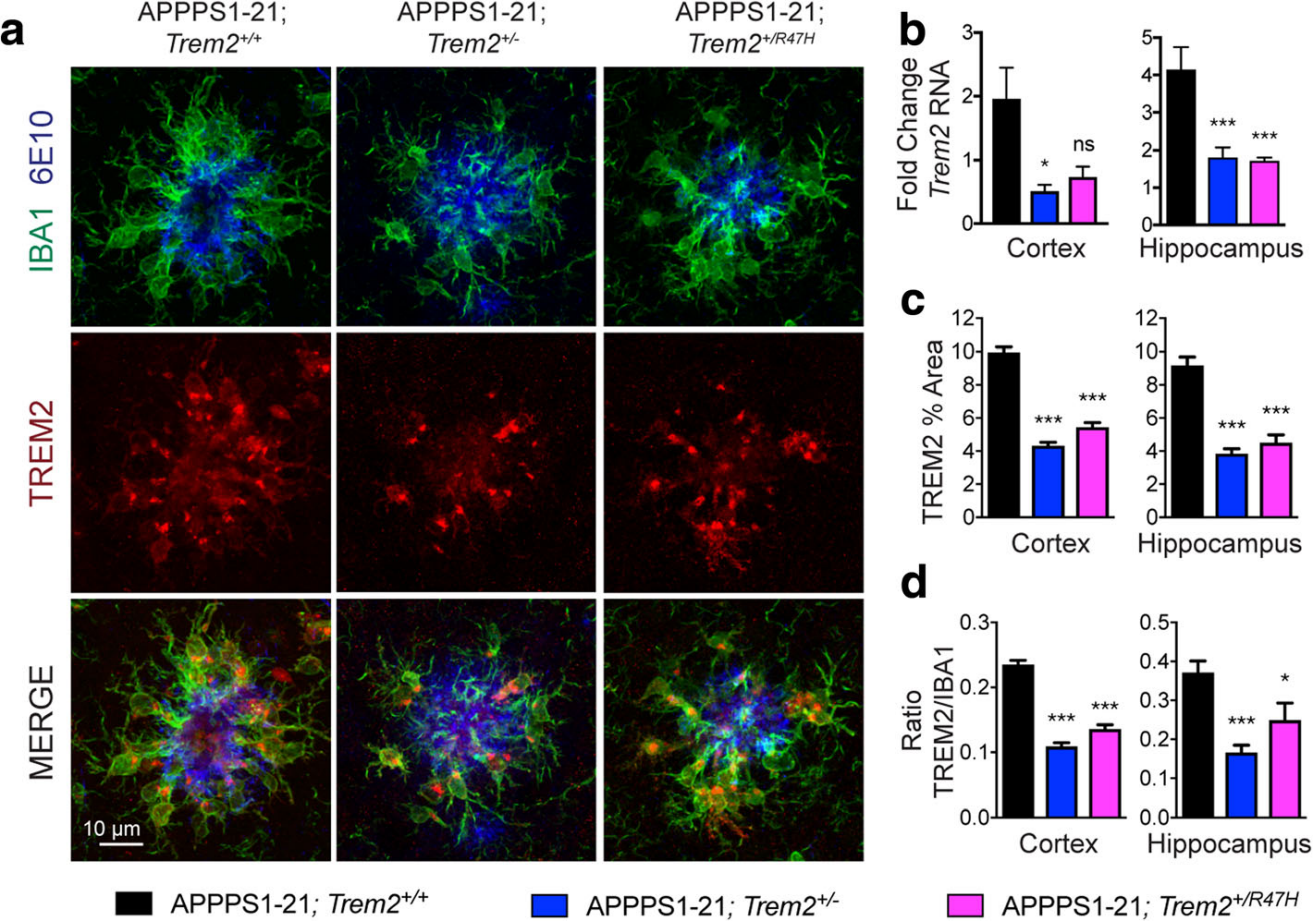
TREM2 expression is significantly reduced in AD mice expressing the *Trem2* R47H variant. **a** Immunohistochemistry was used to identify myeloid cells (IBA1, green), plaques (6E10, blue), and TREM2 (red). **b** *Trem2* RNA levels were assessed in cortical and hippocampal lysates from APPPS1–21;*Trem2*^+/+^ (*n* = 14), APPPS1–21;*Trem2*^+/−^ (*n* = 13), and APPPS1–21;*Trem2*^*+/R47H*^ (*n* = 10) mice. Data are presented as fold change normalized gene expression relative to *Trem2*^+/+^ mice (*n* = 9). **c** Images were quantified to assess TREM2-immunoreactive area and (**d**) the ratio of TREM2 to IBA1 immunoreactive area around plaques (n = 10–13 mice / genotype). Data are presented as mean ± SEM. *p < 0.05; ***p < 0.001; ns - not significant. Representative images are from the cortex

To determine whether the *Trem2* R47H variant affects *Trem2* expression, we evaluated *Trem2* RNA levels in the brains of *Trem2*^+/+^, and *Trem2*^+/R47H^ mice and found a significant 42% decrease in *Trem2* RNA in *Trem2*^+/R47H^ mice compared to *Trem2*^+/+^ mice (Additional file 2: Figure S1C). This suggests that the *Trem2* R47H variant impairs TREM2 expression when endogenous regulation of its expression is maintained, an important consideration when interpreting previous in vitro studies in which *Trem2* R47H expression is induced at WT levels. A significant reduction was observed in *Trem2* expression in APPPS1–21;*Trem2*^+/R47H^ mice compared to APPPS1–21;*Trem2*^+/+^ mice (64% in the hippocampus), similar to the levels observed in APPPS1–21;*Trem2*^*+/−*^ mice (Fig. 1b, Additional file 2: Figure S1D). This reduction in *Trem2* expression in the context of AD suggests that, in addition to reducing baseline *Trem2* expression, the *Trem2* R47H variant may also impair upregulation of *Trem2* expression in response to AD pathology.

As TREM2 protein expression in the AD brain is primarily upregulated on plaque-associated myeloid cells [9, 13], we next evaluated how TREM2 expression was affected in this cell population. Similar to the observed reductions in *Trem2* RNA, the *Trem2* R47H variant resulted in a significant reduction in plaque-associated TREM2 protein expression in APPPS1–21; *Trem2*^+/R47H^ mice compared to APPPS1–21; *Trem2*^+/+^ mice (45% reduction in the cortex and 51% reduction in the hippocampus), similar to the levels observed in APPPS1–21; *Trem2*^*+/−*^ mice (Fig. 1a and c). This reduction in plaque-associated TREM2 protein expression could be due to a reduction in cellular TREM2 expression or due to a reduction in the number of plaque-associated myeloid cells. We found that altered TREM2 expression was not solely due to changes in the presence of myeloid cells around plaques, as TREM2 percent area was still significantly reduced when normalized to the myeloid cell marker IBA1 (Fig. 1d). Together, these data suggest that the *Trem2* R47H variant reduces TREM2 expression in the context of AD.

**Fig. 2:**
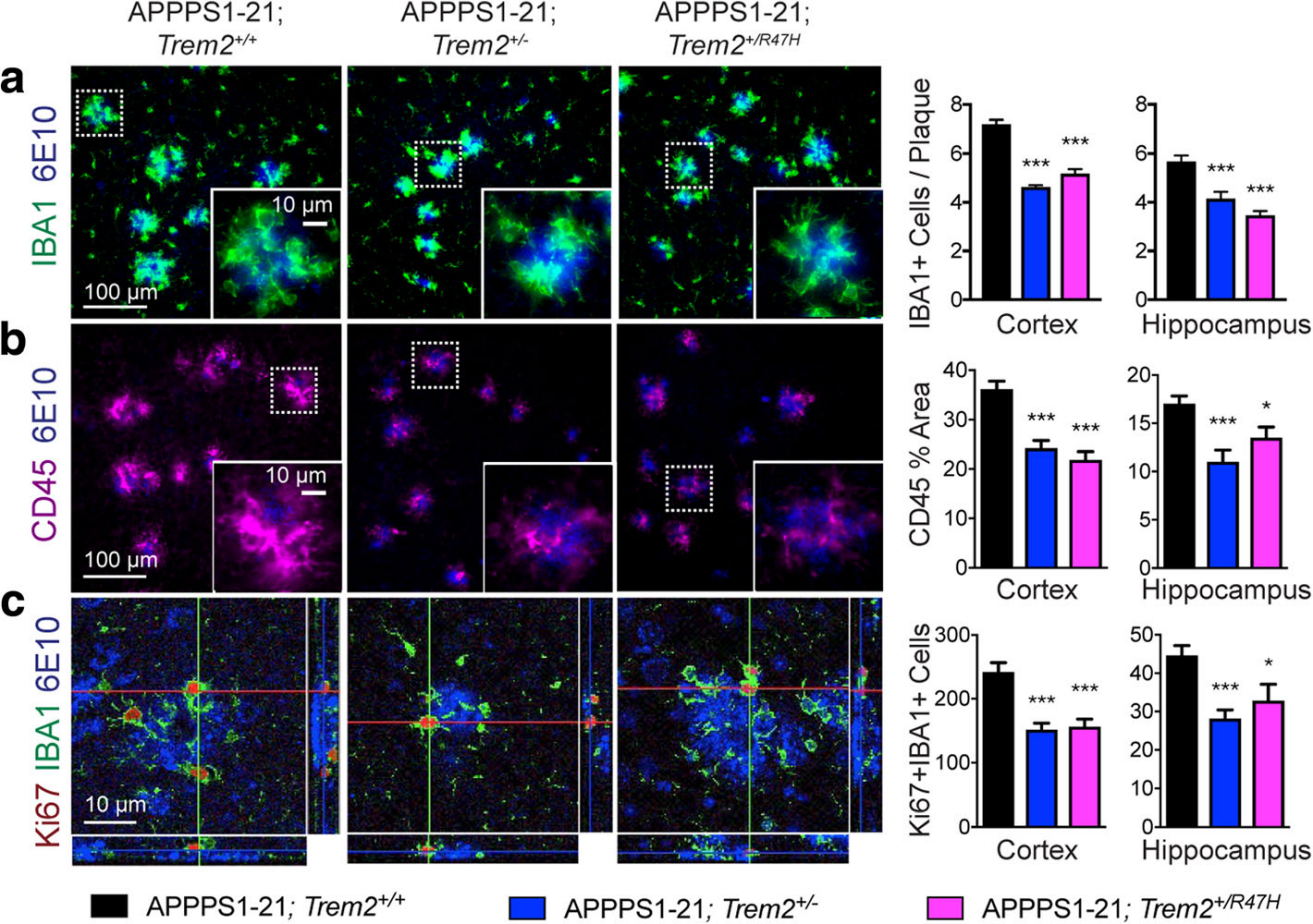
Plaque-associated myeloid cells are reduced in mice expressing the *Trem2* R47H variant. **a** Immunohistochemistry was used to quantify the number of myeloid cells (IBA1, green) around plaques (6E10, blue). **b** Cells expressing high levels of CD45 (magenta) around plaques (6E10, blue) were identified by immunohistochemistry and the percent CD45-positive area per plaque was quantified. **c** Proliferating (Ki67-positive, red) myeloid cells (IBA1-positive, green) were quantified across the entire cortex and hippocampus from one medial and one lateral section. Data from APPPS1–21; *Trem2*^*+/+*^ (*n* = 8), APPPS1–21;*Trem2*^*+/−*^ (*n* = 8), and APPPS1–21;*Trem2*^*+/R47H*^ (*n* = 10) mice are represented as mean ± SEM. *p < 0.05; ** *p* < 0.01; ***p < 0.001; ns - not significant. Representative images are from the cortex

It has been consistently reported that *Trem2* deficiency leads to a specific reduction in accumulation of myeloid cells around plaques, while not significantly affecting non-plaque-associated myeloid cell number [9–13, 28, 29]. To assess whether the *Trem2* R47H variant confers a similar phenotype, we examined the number of IBA1 positive cells around plaques. We found a significant reduction in the number of plaque-associated myeloid cells in APPPS1–21; *Trem2*^+/R47H^ mice compared to APPPS1–21; *Trem2*^+/+^ mice (37% reduction in the cortex and 39% in the hippocampus), at levels comparable to APPPS1–21; *Trem2*^*+/−*^ mice (Fig. 2a), which was consistent across plaque size (Additional file 3: Figure S2A). Thus, AD mice expressing the *Trem2* R47H variant exhibit an impairment in myeloid cell accumulation around plaques consistent with a loss of TREM2 function.

We previously reported that *Trem2* deficiency results in preferential loss of CD45^hi^-expressing myeloid cells around plaques [10, 13]. Canonically, CD45^hi^ has been used to identify peripherally derived myeloid cells [30], though we cannot exclude the possibility that these cells represent a phenotypically distinct subset of reactive microglia. Regardless of their provenance, TREM2 has been shown to be required for accumulation of this cell population in the AD brain. We found a significant reduction in the area of high CD45 immunoreactivity around plaques in APPPS1–21; *Trem2*^+/R47H^ mice relative to APPPS1–21; *Trem2*^+/+^ mice (33% reduction in cortex, 21% in hippocampus), comparable to the reduction observed in APPPS1–21; *Trem2*^+/−^ mice (Fig. 2b). Similar to what has been observed with *Trem2* deficiency, our findings suggest the *Trem2* R47H variant preferentially reduces accumulation of myeloid cells expressing high levels of CD45 around plaques.

The *Trem2* R47H variant could also contribute to reduced myeloid cell number around plaques by increasing myeloid cell death or decreasing myeloid cell proliferation [10, 12]. We only rarely observed cleaved caspase-3 positive myeloid cells across genotypes, so we were unable to assess how the *Trem2* R47H variant affected myeloid cell death. However, proliferation of myeloid cells, assessed using immunohistochemistry to identify Ki67+ IBA1+ cells, was reduced in APPPS1–21; *Trem2*^+/R47H^ mice compared to APPPS1–21; *Trem2*^+/+^ mice (35% reduction in cortex, 27% in hippocampus), similar to APPPS1–21; *Trem2*^+/−^ mice (Fig. 2c). These findings suggest the *Trem2* R47H variant reduces plaque associated myeloid cells, at least in part, through reducing myeloid cell proliferation.

Recent work suggests that TREM2 is required for the myeloid cell-mediated inflammatory response in AD [28, 31]. Therefore, we wanted to assess whether the *Trem2* R47H variant would also impair the inflammatory response to AD pathology. Relative to controls, we detected a significant increase in the RNA levels of *Arg1*, *Ym1*, and *Fizz1*, similar to our previous observations in *Trem2* deficient AD mice (Additional file 3: Figure S2B) [13]. Interestingly, we also observed a significant increase in *IL-6* in mice with the *Trem2* R47H variant. None of these cortical gene expression changes were evident in APPPS1–21; *Trem2*^*+/−*^ mice. There were also significant increases in *Fizz1* and *IL-6* in hippocampal lysates from APPPS1–21; *Trem2*^*+/R47H*^ and APPPS1–21; *Trem2*^*+/−*^ mice relative to APPPS1–21; *Trem2*^*+/+*^ controls (Additional file 3: Figure S2C). A more detailed analysis will be required to fully address the role of *Trem2* R47H on inflammatory responses in AD.

### *Trem2* R47H reduces compact plaque number

**Fig. 3:**
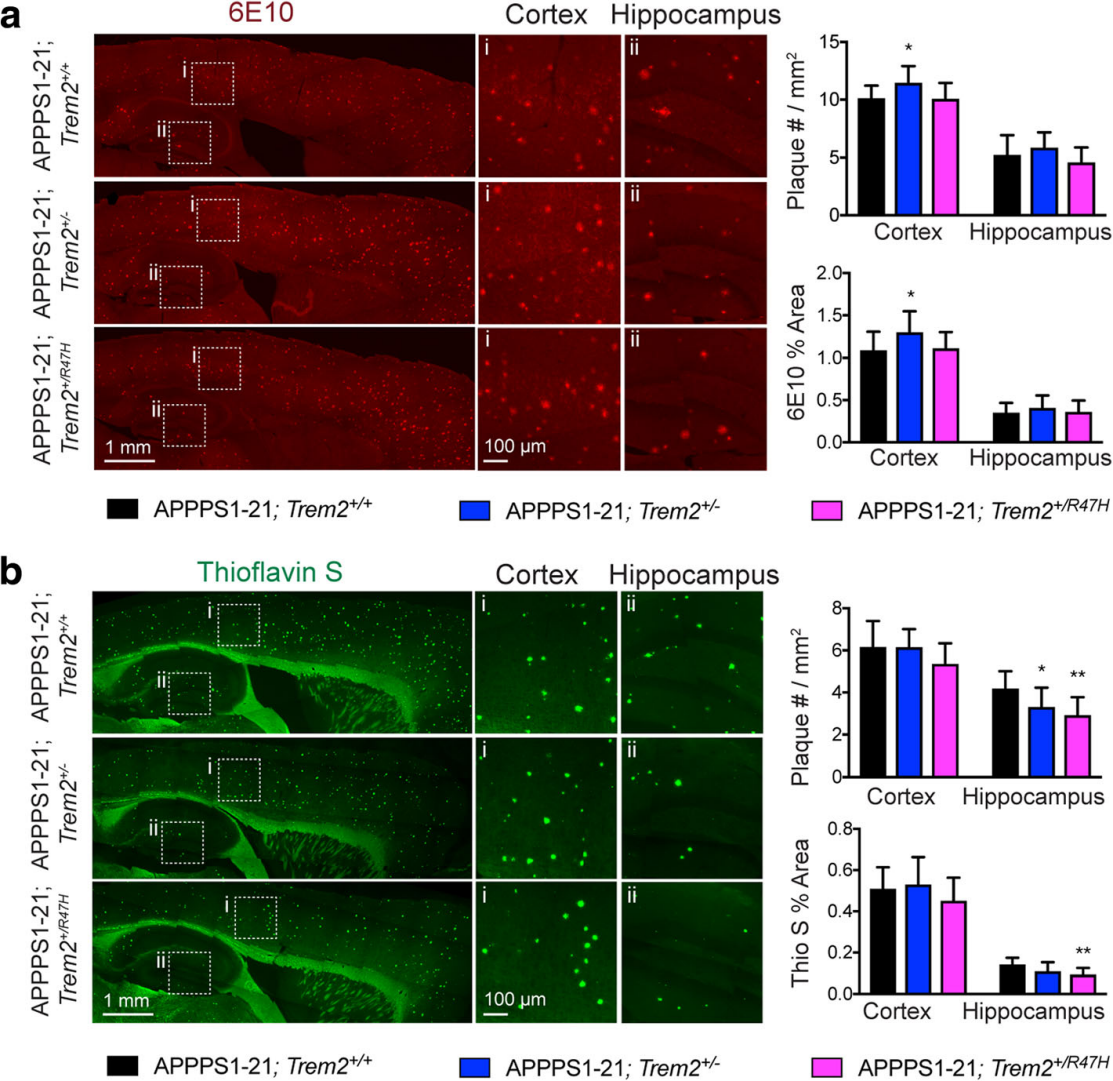
Compact plaque number is specifically reduced in mice expressing the *Trem2* R47H variant. Quantification of plaque burden was performed in APPPS1–21;*Trem2*^*+/+*^ (*n* = 15), APPPS1–21;*Trem2*^*+/−*^ (*n* = 13), and APPPS1–21;*Trem2*^*+/R47H*^ (*n* = 10) mice by (**a**) measuring 6E10 (red) and (**b**) Thioflavin S (green) positive plaque number and percent area across three cortical and one hippocampal region from 10 to 12 sagittal sections. Higher magnification of cortical (i) and hippocampal (ii) regions are shown. Data are presented as mean ± SEM. *p < 0.05; **p < 0.01

Reduced accumulation of myeloid cells around plaques due to loss of TREM2 has previously been shown to result in changes in plaque deposition [10, 11, 13]. *Trem2* deficiency alters plaque burden in a disease progression-dependent manner, increasing plaque accumulation at advanced disease stages, but reducing plaque accumulation early in disease [10]. Consistent with earlier plaque deposition in the cortex relative to the hippocampus in APPPS1–21 mice, at 4 months of age, previous studies found a reduction in amyloid accumulation in the hippocampus with *Trem2* deficiency, but no significant differences in the cortex [13]. These changes occurred independent of changes in AD transgene expression, which we also found were unaltered in mice expressing the *Trem2* R47H variant [10] (Additional file 4: Figure S3A). In order to assess whether the *Trem2* R47H variant modifies total plaque burden, we measured the number and percent area of 6E10 positive plaques. While a modest increase in total cortical plaque number and percent area were noted in APPPS1–21;*Trem2*^+/−^ mice compared to APPPS1–21;*Trem2*^+/+^ mice, no difference in 6E10 positive plaque number or percent area was observed in APPPS1–21; *Trem2*^+/R47H^ mice (Fig. 3a). Previous studies have demonstrated that *Trem2* deficient mice exhibit a shift in plaque structure, from compact, fibrillar plaques to diffuse plaques [9]. To determine whether the *Trem2* R47H variant affected the relative abundance of these different plaque types, we quantified the number and percent area of fibrillar, thioflavin S positive plaques. A significant reduction in thioflavin S positive plaque number (31%) and percent area (36%) were observed in the hippocampus of APPPS1–21; *Trem2*^+/R47H^ mice compared to APPPS1–21; *Trem2*^+/+^ mice (Fig. 3b).

To assess whether this shift in plaque morphology was due to alterations in the presence of different Aβ species, we used ELISAs to assess soluble and insoluble Aβ_1–40_ and Aβ_1–42._ We observed a significant decrease in soluble Aβ_1–40_ in the cortex of APPPS1–21; *Trem2*^+/R47H^ mice compared to APPPS1–21;*Trem2*^+/+^ mice (Additional file 4: Figure S3B), and thus an increased ratio of Aβ42/40 (Additional file 4: Figure S3C) in APPPS1–21; *Trem2*^*+/R47H*^ mice relative to APPPS1–21; *Trem2*^*+/+*^ controls. We also observed a significant increase in soluble Aβ_1–40_ in the hippocampus of APPPS1–21; *Trem2*^+/−^ mice compared to APPPS1–21; *Trem2*^+/+^ mice but no significant changes in other Aβ species in mice expressing the *Trem2* R47H variant. Together, these data suggest that changes in the relative abundance of these species are not the primary contributor to changes in plaque structure in mice with the *Trem2* R47H variant. However, fibrillar plaques are specifically reduced in mice bearing the *Trem2* R47H variant, consistent with results from *Trem2* deficient mice and human carriers of *TREM2* R47H [9].

### *Trem2* R47H significantly increases plaque-associated neuritic dystrophy

**Fig. 4:**
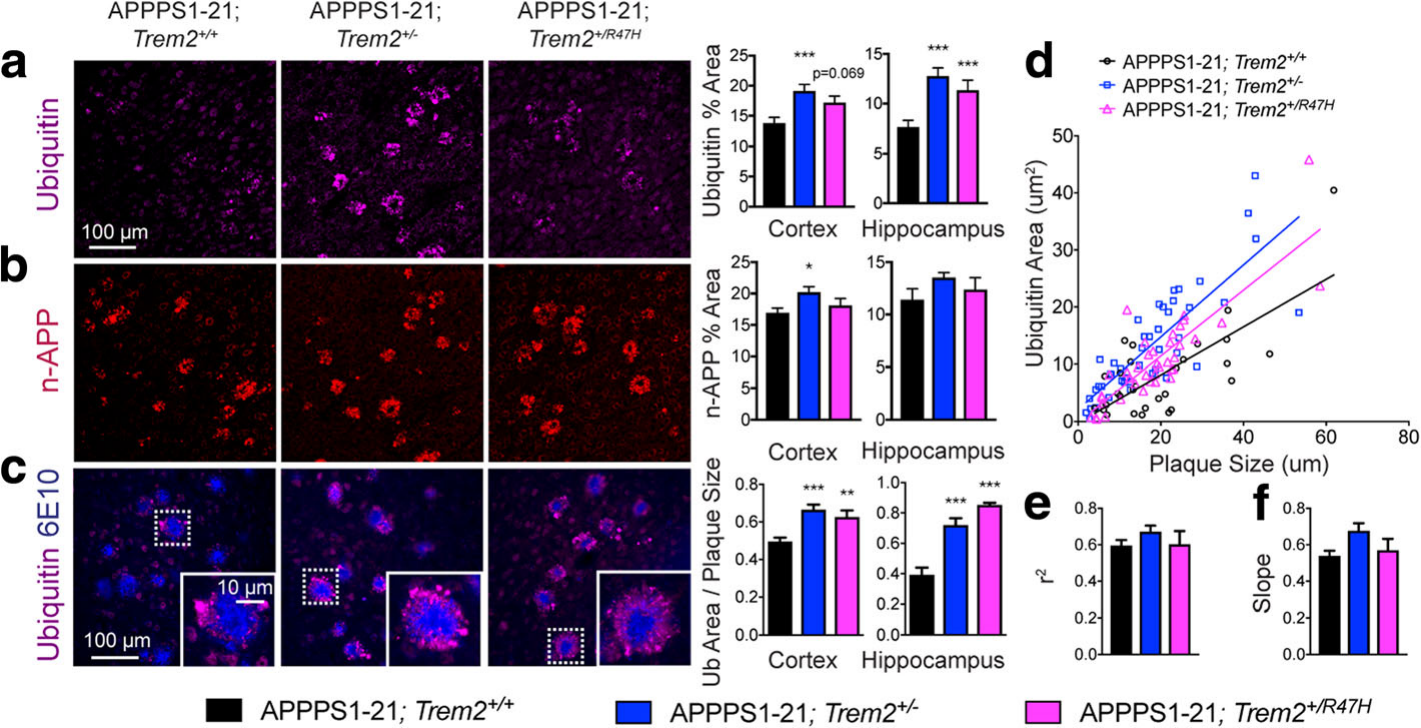
Neuritic dystrophy is increased in APPPS1–21 mice expressing the *Trem2* R47H variant. **a** Immunohistochemistry was used to quantify dystrophic neurites in APPPS1–21;*Trem2*^+/+^ (*n* = 16), APPPS1–21;*Trem2*^+/−^ (n = 15), and APPPS1–21;*Trem2*^*+/R47H*^ (*n* = 10) mice by measuring (**a**) ubiquitin (magenta) and (**b**) N-terminal APP (n-APP, red) % area across the cortex and hippocampus. **c** Dystrophic neurite area (ubiquitin, magenta) normalized to plaque (6E10, blue) size was assessed in APPPS1–21;*Trem2*^*+/+*^ (*n* = 16), APPPS1–21;*Trem2*^*+/−*^ (*n* = 15), and APPPS1–21;*Trem2*^*+/R47H*^ (*n* = 10) mice. **d** The correlation between ubiquitin positive area and plaque size was plotted for one representative animal per *Trem2* genotype and (**e**) r^2^ and (**f**) slope for the linear best fit lines were calculated. Data are presented as mean ± SEM. *p < 0.05; **p < 0.01; ***p < 0.001

Damage to axons and dendrites in the vicinity of plaques, termed neuritic dystrophy, is thought to contribute to cognitive impairment in AD [32] and is reported to be enhanced in AD mice lacking *Trem2* and humans carrying an R47H allele [9, 12]. To determine whether the changes in plaque structure observed in AD mice with the *Trem2* R47H variant similarly affected neuritic dystrophy, we next analyzed plaque-associated N-terminal APP (n-APP), which is elevated in dystrophic neurites due to impaired anterograde transport, and ubiquitin, which is increased in response to cellular stress and protein dyshomeostasis. A significant 33% increase in ubiquitin percent area around plaques was observed in the hippocampus of APPPS1–21;*Trem2*^+/R47H^ mice compared to APPPS1–21;*Trem2*^+/+^ mice, similar to levels in APPPS1–21;*Trem2*^*+/−*^ mice (Fig. 4a). Comparable trends in neuritic dystrophy were observed using the additional dystrophic neurite marker n-APP, though the changes with *Trem2* genotype were only significant in the cortex of APPPS1–21;*Trem2*^*+/−*^ mice relative to APPPS1–21;*Trem2*^*+/+*^ mice (Fig. 4b). The ratio of ubiquitin area to plaque size was significantly increased in APPPS1–21;*Trem2*^+/R47H^ mice compared to APPPS1–21;*Trem2*^+/+^ mice (Fig. 4c). Interestingly, the correlation between plaque size and ubiquitin positive area was preserved across genotypes (Fig. 4d and e) and there was a trend toward an increase in the slope of the best fit line between ubiquitin positive area and plaque size in mice with the *Trem2* R47H variant (Fig. 4f). This indicates that larger plaques may be even more strongly affected by the loss of TREM2 function. Together, our data demonstrate overall enhanced neuritic dystrophy with the *Trem2* R47H variant, when normalized to plaque size, suggesting a possible mechanism by which the variant could increase synaptic loss and neuronal dysfunction, and ultimately confer AD risk.

## Discussion

In order to investigate how the *Trem2* R47H variant affects TREM2 function and AD pathology, we developed a CRISPR/Cas9 knock-in of the R47H variant into the mouse *Trem2* gene. Because this approach maintains endogenous regulation of TREM2 expression, we were able to determine that expression of one copy of the R47H variant reduces *Trem2* expression in a wild-type background and further impairs upregulation of *Trem2* expression in an AD mouse model. This finding differs from a previous study that found no changes in TREM2 expression in postmortem tissue from human AD patients heterozygous for the *TREM2* R47H variant [33]. While many factors could contribute to this discrepancy, *Trem2* levels are known to change throughout disease progression [13], and our study evaluates *Trem2* changes at a relatively early stage in pathology in the APPPS1–21 model, while the postmortem samples are from humans at a late stage in disease. It will be interesting to assess in future studies whether *Trem2* levels are differentially affected by the R47H variant throughout disease progression.

Importantly, this finding also merits consideration when interpreting studies of TREM2 R47H function in vitro, which have all used systems where *Trem2* R47H expression is maintained at WT levels, and the recent evaluation of *Trem2* R47H variant function using BAC transgenics where *Trem2* was overexpressed. It is possible that the observed loss-of-function phenotypes may arise, at least in part, through reduced expression of TREM2. Furthermore, by knocking the R47H variant into the mouse *Trem2* gene, we maintain the appropriate interaction of mouse TREM2 with its endogenous ligands and signaling molecules. However, despite a high degree of homology between human *TREM2* and mouse *Trem2* genes, it is possible that the R47H variant affects human TREM2 differently than it affects mouse TREM2 structure and function. This caveat of our approach is addressed by complementary work using a BAC to express human TREM2 R47H in *Trem2*-deficient AD model [21]. Notably, our CRISPR/Cas9 knock-in approach and their BAC transgenic yield comparable results in myeloid cell accumulation around plaques, Together, these findings suggest that the *Trem2* R47H variant confers phenotypes consistent with loss of TREM2 function in a mouse model of AD-like amyloid deposition.

AD mice expressing the *Trem2* R47H variant exhibit reduced plaque-associated myeloid cells. We find that this is, in part, due to reduced proliferation. In addition, we demonstrate a selective reduction in plaque-associated cells expressing high levels of CD45 in mice expressing the *Trem2* R47H variant. It remains unclear whether the reduction in myeloid cell number represents impaired recruitment or survival of peripherally derived macrophages in the AD brain or diminished phenotypic conversion of resident microglia to adopt expression of this marker. Other possible mechanisms may also contribute to the reduction of myeloid cells around plaques in mice expressing the *Trem2* R47H variant, including deficits in myeloid cell migration [34] and survival [11].

The alterations in myeloid cell accumulation are also reflected by changes in inflammation-related gene expression. While changes in hippocampal gene expression are largely similar between APPPS1–21;*Trem2*^*+/R47H*^ mice and APPPS1–21;*Trem2*^*+/−*^ mice, in the cortex, increases in mRNA levels of *Arg1*, *Fizz1*, *Ym1* and *IL-6* are specific to mice expressing the *Trem2* R47H variant. This demonstrates that there are some functional measures in which the R47H variant does not completely phenocopy loss of one copy of *Trem2*. These differences in cortical gene expression between APPPS1–21;*Trem2*^*+/R47H*^ and APPPS1–21;*Trem2*^*+/−*^ mice are not reflected in differences in the other myeloid cell phenotypes or features of pathology assessed in this manuscript. Additional experiments will be required to fully address whether these region-specific alterations in gene expression relate to other meaningful differences in myeloid cell function and pathology.

Our data show that the *Trem2* R47H variant does not alter 6E10 positive plaque burden, but does reduce compact, thioflavin S positive plaques, suggesting that the changes in myeloid cell function mediated by the *Trem2* R47H variant result in altered plaque structure. Yuan et al. suggested that this could be due to impaired accumulation of myeloid cells around plaques, which may normally limit plaque growth. However, it has also been shown that TREM2 influences the phagocytic activity of myeloid cells, which could also contribute to changes in plaque structure.

It has been previously postulated that myeloid cells form a barrier around plaques, protecting surrounding neurites from damaging Aβ species [35], leading to the prediction that impaired association of myeloid cells with plaques would increase neuritic dystrophy. Indeed, studies have previously shown enhanced neuritic dystrophy with reduced myeloid cell plaque coverage in AD mice deficient for *Trem2*, and in AD patients carrying the *TREM2* R47H variant [9, 12]. Consistent with these findings, we observed an increase in dystrophic neurites, relative to plaque size, in mice expressing the *Trem2* R47H variant. However, it has also been shown that larger plaques typically have less microglial coverage and more neuritic dystrophy. Thus, we expected that reduced myeloid cell accumulation around plaques with changes in *Trem2* genotype would preferentially increase neuritic dystrophy around small plaques, and have less impact on larger plaques, since these plaques already exhibit little myeloid cell coverage. In contrast, however, we find that dystrophic neurite area correlated just as strongly with plaque size in both APPPS1–21;*Trem2*^+/R47H^ and APPPS1–21;*Trem2*^+/−^ mice. Furthermore, there was a trend toward an increase in the slope between dystrophic neurite area and plaque size in APPPS1–21;*Trem2*^+/R47H^ and APPPS1–21;*Trem2*^+/−^ mice relative to controls, suggesting that larger plaques may be even more strongly affected by the loss of TREM2 function, and consequently reduced accumulation of plaque-associated myeloid cells. Together, these data are suggestive of additional roles for TREM2 in modulating neuritic dystrophy other than limiting access of plaque species to surrounding neurites. These findings suggest that TREM2 may be involved in other mechanisms of dystrophic neurite formation, or perhaps more likely, given its demonstrated role of phagocytosis in vitro, in the clearance of these dystrophic neurites [5]. It will be important to determine whether the enhanced neuritic dystrophy also correlates with neurodegeneration and cognitive deficits.

A central question arising from this work is how the changes observed in our study relate to the approximate three-fold elevation in AD risk in heterozygous carriers of the *TREM2* R47H variant. Our data demonstrate that the *Trem2* R47H variant impairs TREM2 function, in part by reducing TREM2 expression. This results in a reduced myeloid cell response to AD pathology, and increased neuritic dystrophy. Our results highlight the important functional roles of myeloid cells in AD pathogenesis and progression, and suggest that enhancing TREM2 signaling may be beneficial in the context of sporadic AD. In addition, because the *TREM2* R47H variant confers risk for other neurodegenerative diseases, this study also provides a basis for understanding important myeloid cells functions and provides potential avenues for therapeutic targets in other disease contexts. Collectively, understanding the mechanism by which the *Trem2* R47H variant affects myeloid cell function and pathology across multiple disease models promises to decipher common mechanisms by which myeloid cells modulate neurodegenerative disease pathology.

## Conclusions

In summary, our findings indicate that the Alzheimer’s disease-associated *Trem2* R47H variant confers a loss of TREM2 function, impairing myeloid cell responses to pathology. This results in a reduction in TREM2 expression, myeloid cell proliferation, reduced compact plaque burden and enhanced neuritic dystrophy in an Alzheimer’s disease mouse model. These findings were comparable to AD mice lacking one copy of *Trem2.*

## Additional files

Additional file 1: **Table S1.** Variant calling for APPPS1–21;*Trem2*^*+/R47H*^ mice for the CRISPR predicted off target gene *Rab11fip3*, *Trem2,* and *Trem*-like genes *Treml1*, *Treml2*, and *Treml6*. “0/1” indicates a heterozygous variant and “./.” indicates no variants detected. Variants detected in *Trem2* R47H lines but not APPPS1–21; *Trem2*^+/+^ or *Trem2*^+/+^ mice were considered to be true. (XLSX 14 kb)

Additional file 2: **Figure S1.** (A) The SNP encoding for the arginine-to-histidine missense mutation was knocked into exon 2 of mouse *Trem2* using CRISPR/Cas9 targeting. The sequences for the reference genome, guide RNA (antisense), and homology directed repair (HDR) oligonucleotide containing the AD-associated R47H variant (red) and a silent mutation (blue) to ablate the protospacer adjacent motif (PAM), are indicated. Sanger sequence alignment from a representative *Trem2*^*+/R47H*^ mouse is shown. (B) Comparison of major findings across two independently generated *Trem2* R47H founder lines are shown for APPPS1–21; *Trem2*^+/R47H^ mice from line R104 (*n* = 7) and line R1019 (*n* = 3). (C) RNA levels of *Trem2* were assessed in cortical lysates from *Trem2*^+/+^ (*n* = 9), and *Trem2*^*+/R47H*^ (*n* = 10) mice. (D) RNA levels of *Trem2* were assessed in cortical and hippocampal lysates from APPPS1–21;*Trem2*^+/+^ (*n* = 6 females, *n* = 6 males), APPPS1–21;*Trem2*^+/−^ (*n* = 5 females, *n* = 8 males), and APPPS1–21;*Trem2*^*+/R47H*^ (*n* = 5 females, *n* = 5 males) mice. Data are presented as fold change normalized gene expression relative to *Trem2*^+/+^ mice (*n* = 4 females, *n* = 4 males) and were analyzed using a two-way ANOVA. *p < 0.05; ***p < 0.001; ns - not significant. (TIF 9131 kb)

Additional file 3: **Figure S2.** (A) IBA1+ cell number per plaque was assessed relative to plaque size in cortex from APPPS1–21;*Trem2*^+/+^ (*n* = 4), APPPS1–21;*Trem2*^+/−^ (*n* = 6), and APPPS1–21;*Trem2*^*+/R47H*^ (*n* = 4) mice. Data are presented as mean ± SEM. (B) Inflammation-related genes were assessed in cortical and (C) hippocampal lysates from APPPS1–21;*Trem2*^+/+^ (*n* = 15), APPPS1–21;*Trem2*^+/−^ (*n* = 12), and APPPS1–21;*Trem2*^*+/R47H*^ (*n* = 10) mice. Data are presented as fold change normalized gene expression,*p < 0.05, **p < 0.01, ****p < 0.0001. (TIF 9131 kb)

Additional file 4: **Figure S3.** (A) Expression of amyloid precursor protein (*App)* and related genes were assessed in cortical lysates from APPPS1–21;*Trem2*^+/+^ (*n* = 13), APPPS1–21;*Trem2*^+/−^ (*n* = 13), and APPPS1–21;*Trem2*^*+/R47H*^ (*n* = 8) mice. Data are presented as fold change normalized gene expression. (B) ELISAs for Aβ_1–40_ and Aβ_1–42_ and (C) ratio of Aβ_1–42_/Aβ_1–40_ were performed on DEA (soluble) and FA (insoluble) fractions from cortex and hippocampus from APPPS1–21;*Trem2*^*+/+*^ (*n* = 17), APPPS1–21;*Trem2*^*+/−*^ (*n* = 14), and APPPS1–21;*Trem2*^*+/R47H*^ (*n* = 10) mice. Data are presented as fold change normalized protein expression. *p < 0.05, **p < 0.01. (TIF 9131 kb)

## Abbreviations

AD: Alzheimer’s disease
BAC: Bacterial artificial chromosome
CRISPR: Clustered regularly interspaced short palindromic repeats
IBA1: Ionized calcium-binding adapter molecule 1
PAM: Protospacer adjacent motif
SNP: Single nucleotide polymorphism
TREM2: Triggering receptor expressed on myeloid cells 2
