# Supplementary figures and images for: The Trem2 R47H variant confers loss-of-function-like phenotypes in Alzheimer’s disease
Source: Mol Neurodegener. 2018 Jun 1;13:29. doi: 10.1186/s13024-018-0262-8 (PMC5984804; doi:10.1186/s13024-018-0262-8)

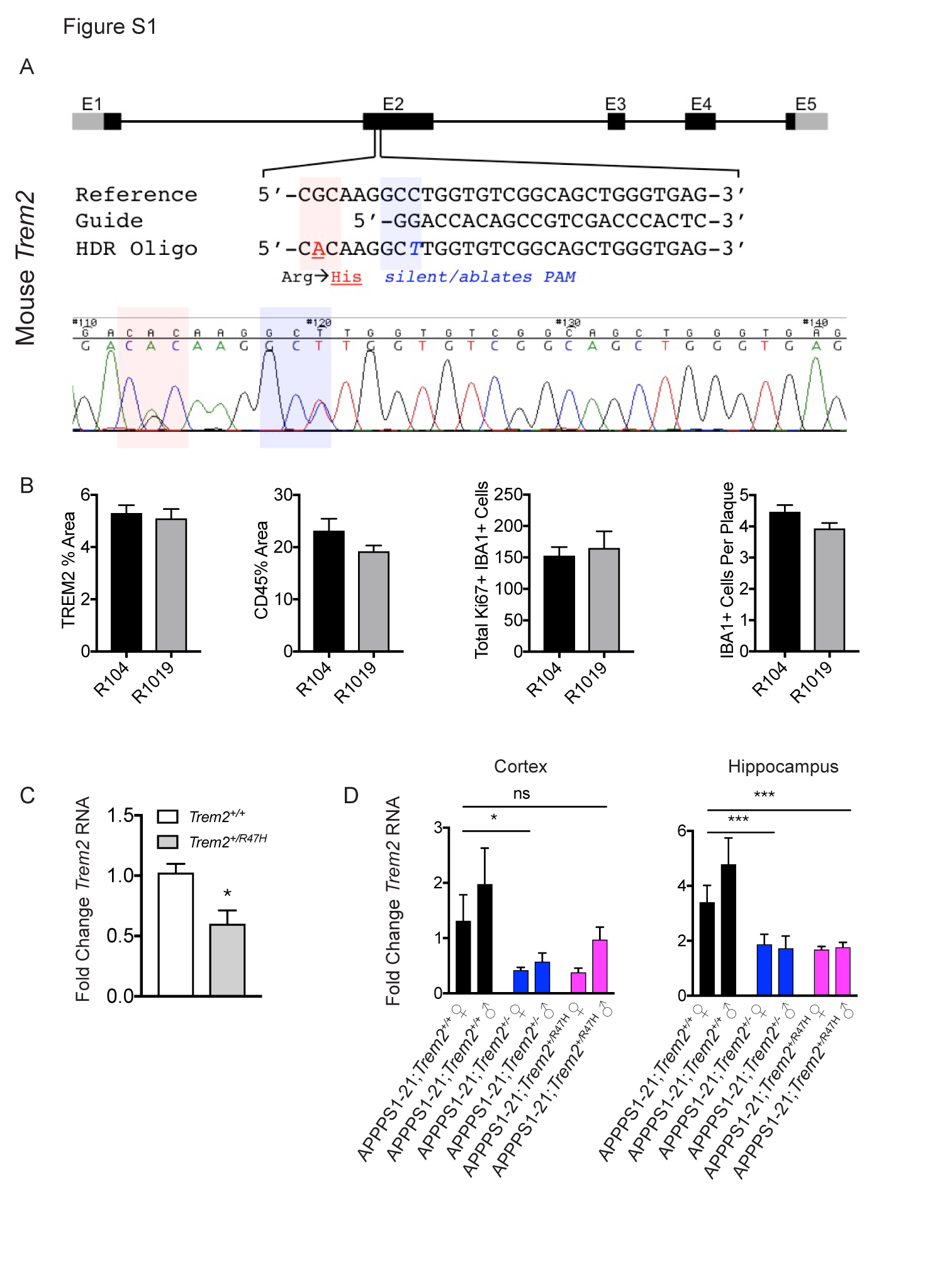

Supplement: Supplementary file 2 — Figure S1. (A) The SNP encoding for the arginine-to-histidine missense mutation was knocked into exon 2 of mouse Trem2 using CRISPR/Cas9 targeting. The sequences for the reference genome, guide RNA (antisense), and homology directed repair (HDR) oligonucleotide containing the AD-associated R47H variant (red) and a silent mutation (blue) to ablate the protospacer adjacent motif (PAM), are indicated. Sanger sequence alignment from a representative Trem2+/R47H mouse is shown. (B) Comparison of major findings across two independently generated Trem2 R47H founder lines are shown for APPPS1–21; Trem2+/R47H mice from line R104 (n = 7) and line R1019 (n = 3). (C) RNA levels of Trem2 were assessed in cortical lysates from Trem2+/+ (n = 9), and Trem2+/R47H (n = 10) mice. (D) RNA levels of Trem2 were assessed in cortical and hippocampal lysates from APPPS1–21;Trem2+/+ (n = 6 females, n = 6 males), APPPS1–21;Trem2+/− (n = 5 females, n = 8 males), and APPPS1–21;Trem2+/R47H (n = 5 females, n = 5 males) mice. Data are presented as fold change normalized gene expression relative to Trem2+/+ mice (n = 4 females, n = 4 males) and were analyzed using a two-way ANOVA. *p < 0.05; ***p < 0.001; ns - not significant. (TIF 9131 kb) [file 13024_2018_262_MOESM2_ESM.tif]

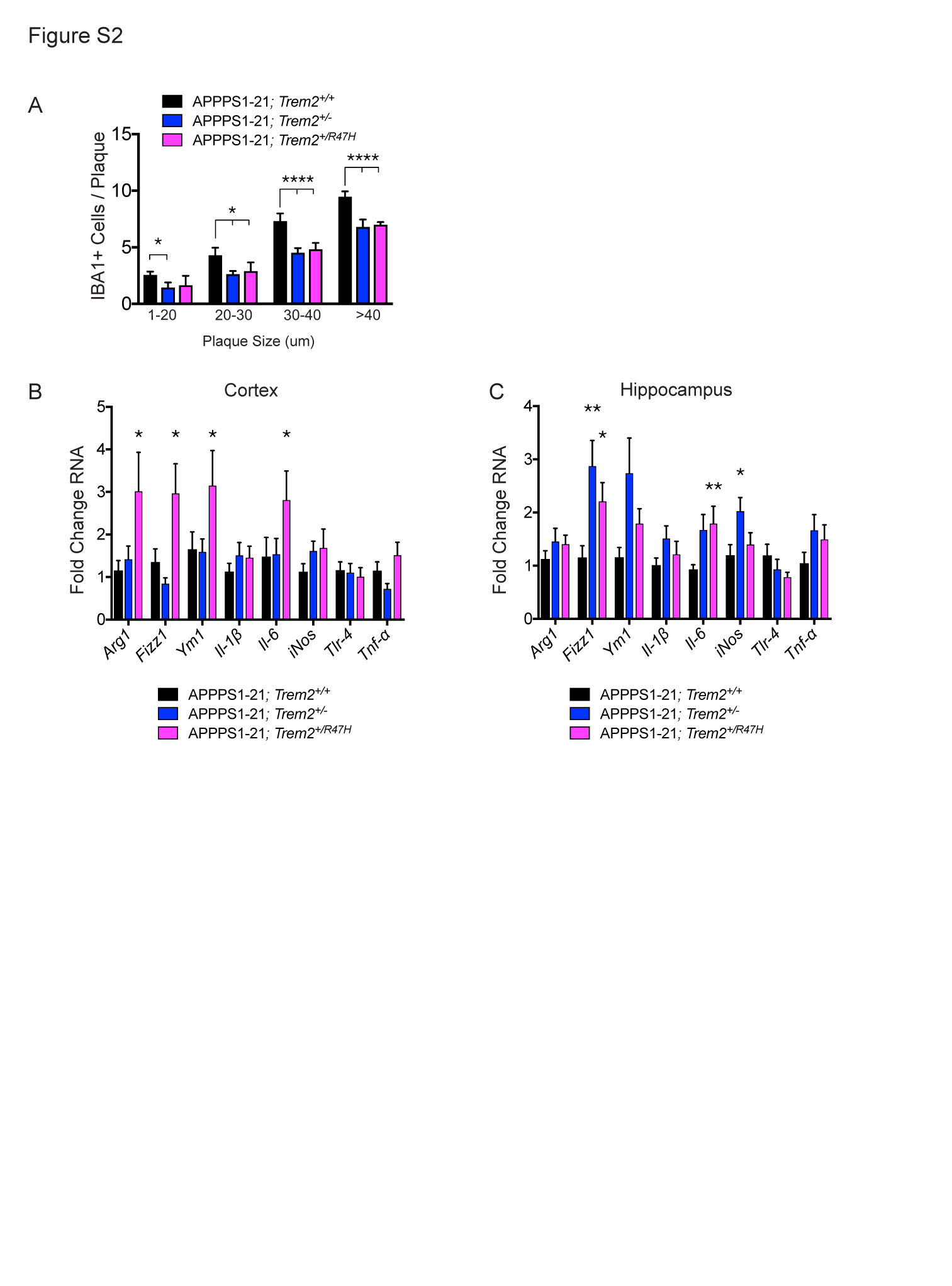

Supplement: Supplementary file 3 — Figure S2. (A) IBA1+ cell number per plaque was assessed relative to plaque size in cortex from APPPS1–21;Trem2+/+ (n = 4), APPPS1–21;Trem2+/− (n = 6), and APPPS1–21;Trem2+/R47H (n = 4) mice. Data are presented as mean ± SEM. (B) Inflammation-related genes were assessed in cortical and (C) hippocampal lysates from APPPS1–21;Trem2+/+ (n = 15), APPPS1–21;Trem2+/− (n = 12), and APPPS1–21;Trem2+/R47H (n = 10) mice. Data are presented as fold change normalized gene expression,*p < 0.05, **p < 0.01, ****p < 0.0001. (TIF 9131 kb) [file 13024_2018_262_MOESM3_ESM.tif]

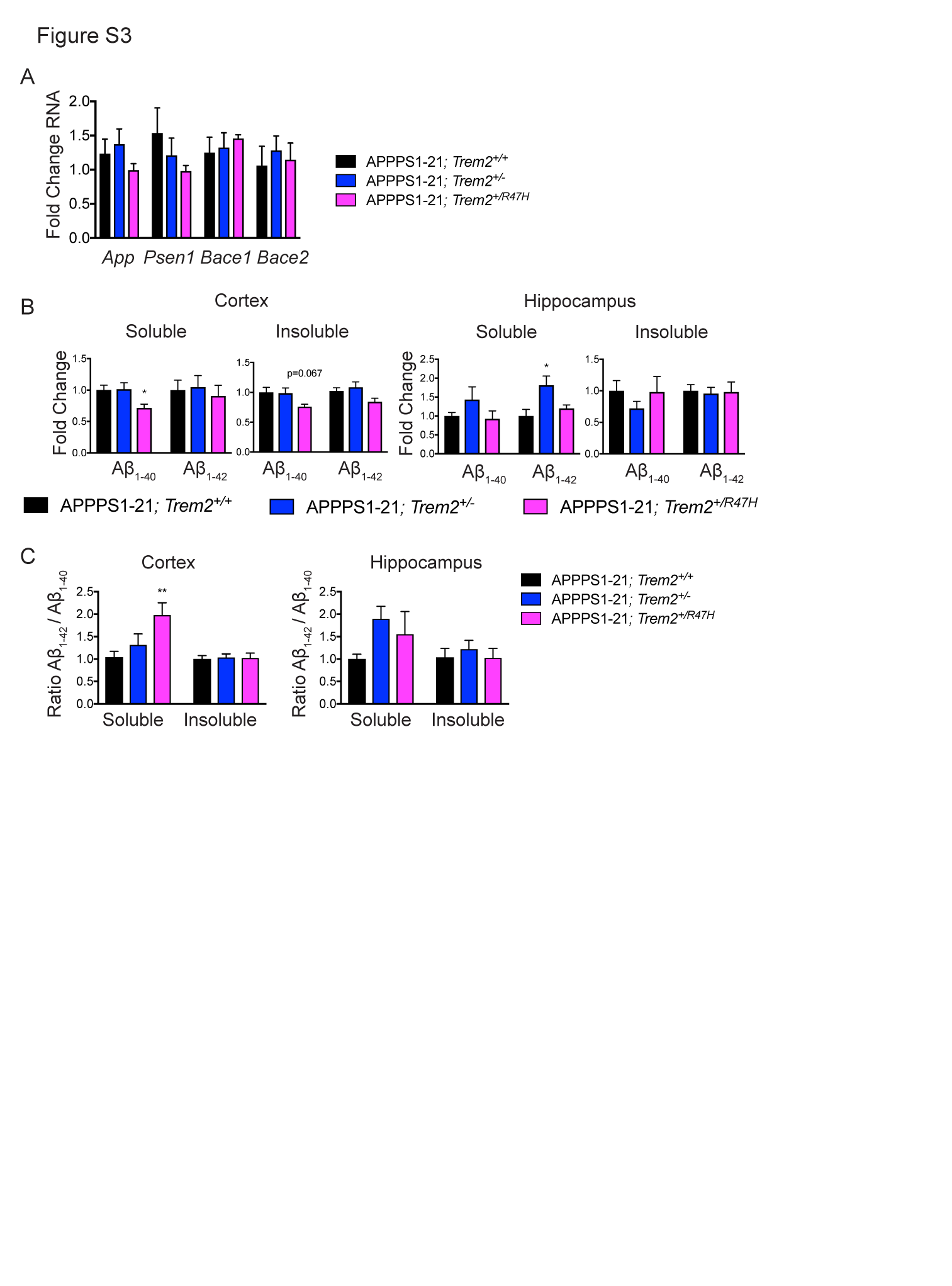

Supplement: Supplementary file 4 — Figure S3. (A) Expression of amyloid precursor protein (App) and related genes were assessed in cortical lysates from APPPS1–21;Trem2+/+ (n = 13), APPPS1–21;Trem2+/− (n = 13), and APPPS1–21;Trem2+/R47H (n = 8) mice. Data are presented as fold change normalized gene expression. (B) ELISAs for Aβ1–40 and Aβ1–42 and (C) ratio of Aβ1–42/Aβ1–40 were performed on DEA (soluble) and FA (insoluble) fractions from cortex and hippocampus from APPPS1–21;Trem2+/+ (n = 17), APPPS1–21;Trem2+/− (n = 14), and APPPS1–21;Trem2+/R47H (n = 10) mice. Data are presented as fold change normalized protein expression. *p < 0.05, **p < 0.01. (TIF 9131 kb) [file 13024_2018_262_MOESM4_ESM.tif]
